# Resting-state BOLD temporal variability of the default mode network predicts spontaneous mind wandering, which is negatively associated with mindfulness skills

**DOI:** 10.3389/fnhum.2025.1515902

**Published:** 2025-01-22

**Authors:** Sara Sorella, Cristiano Crescentini, Alessio Matiz, Minah Chang, Alessandro Grecucci

**Affiliations:** ^1^Department of Languages and Literatures, Communication, Education and Society, University of Udine, Udine, Italy; ^2^Department of Psychology, Sapienza University of Rome, Rome, Italy; ^3^Clinical and Affective Neuroscience Lab, Department of Psychology and Cognitive Sciences, University of Trento, Rovereto, Italy; ^4^Centre for Medical Sciences, University of Trento, Trento, Italy

**Keywords:** mind wandering, mindfulness, temporal variability, default mode network, spontaneous mind wandering, deliberate mind wandering

## Abstract

Mind wandering (MW) encompasses both a deliberate and a spontaneous disengagement of attention from the immediate external environment to unrelated internal thoughts. Importantly, MW has been suggested to have an inverse relationship with mindfulness, a state of nonjudgmental awareness of present-moment experience. Although they are, respectively, associated with increased and decreased activity in the default mode network (DMN), the specific contributions of deliberate and spontaneous MW, and their relationships with mindfulness abilities and resting-state macro networks remain to be elucidated. Therefore, resting-state MRI scans from 76 participants were analyzed with group independent component analysis to decompose brain networks into independent macro-networks and to see which of them predicted specific aspects of spontaneous and deliberate MW or mindfulness traits. Our results show that temporal variability of the resting-state DMN predicts spontaneous MW, which in turn is negatively associated with the *acting with awareness* facet of mindfulness. This finding shows that the DMN is not directly associated with overall mindfulness, but rather demonstrates that there exists a close relationship between DMN and MW, and furthermore, that the involvement of mindfulness abilities in this dynamic may be secondary. In sum, our study contributes to a better understanding of the neural bases of spontaneous MW and its relationship with mindfulness. These results open up the possibility of intervening on specific aspects of our cognitive abilities: for example, our data suggest that training the mindfulness facet *acting with awareness* would allow lessening our tendency for MW at inopportune times.

## 1 Introduction

Mind wandering (MW) is a heterogeneous construct, defined as a shift of attention from the present moment, current task, or external stimuli to unrelated thoughts, and the convergence of its different definitions highlight several non-mutually exclusive dimensions such as intentionality, stimulus-dependency, task-relatedness, and thought constraints (Christoff et al., 2016; Feruglio et al., 2021). MW is a basic aspect of human cognition that engages people’s minds during 30–50% of their waking hours (Kane et al., 2007; Killingsworth and Gilbert, 2010). MW includes a deliberate process that entails cognitive control through executive brain functions, and a spontaneous process that involves affective salience and automatic mental habits (Feruglio et al., 2021). MW has been described through different perspectives (see Ottaviani and Couyoumdjian, 2013; Seli et al., 2014; Smallwood and Andrews-Hanna, 2013), ranging from negative characteristics, such as rumination, depression and unhappiness (Killingsworth and Gilbert, 2010; van Vugt et al., 2018; Xu et al., 2024), to positive characteristics, such as creativity (Baird et al., 2012) and future planning (Baird et al., 2011). Therefore, distinguishing different forms of MW as research variables allows making an important differentiation among the various, and even opposing, phenomenology of this construct (Cantone et al., 2021).

Its neural underpinnings are closely associated with the default-mode network (DMN), which includes the medial prefrontal cortex, posterior cingulate cortex, and inferior parietal lobule (Raichle et al., 2001). The DMN shows increased activity during MW episodes (Christoff et al., 2009; Christoff et al., 2016; Mason et al., 2007). It has also been associated with MW characteristics (Feruglio et al., 2020), such as semantic processing (Binder et al., 2009), task-unrelated thoughts (Andrews-Hanna et al., 2014; Axelrod et al., 2017; Fox et al., 2015), unconstrained thoughts (Christoff et al., 2016), and self-related processing (Buckner and Carroll, 2007; Moran et al., 2006). Alterations of DMN have been linked to many psychological disorders, such as borderline (Grecucci et al., 2022; Grecucci et al., 2023; Langerbeck et al., 2023; Xiao et al., 2024) and narcissistic personality disorders (Jornkokgoud et al., 2023; Jornkokgoud et al., 2024) anxiety (Baggio et al., 2023), schizophrenia and bipolar disorder (Sorella et al., 2019), which shows alterations in spontaneous thought processes and MW (e.g., Christoff et al., 2016).

Further studies explored how we can cultivate the ability to recognize and regulate MW episodes (Feruglio et al., 2020), for instance, through mindfulness practices. Mindfulness can be defined as a mental state of nonjudgmental present-moment awareness, which encompasses self-regulation of attention and a particular orientation of curiosity and acceptance of experience (Bishop et al., 2004). Evidence from behavioral (Mrazek et al., 2013), physiological (Matiz et al., 2019) and neuroscience research (Grecucci et al., 2015) supports that MW and mindfulness engage at least partially opposed psychological and brain processes. Mindfulness may alter the DMN, promoting a shift from self-referential thinking to a more balanced state of present-moment awareness (Brewer et al., 2011; Tang et al., 2015). Decreased DMN activation (Brewer et al., 2011; Garrison et al., 2015) and gray matter alterations (Hölzel et al., 2011) associated with mindfulness practice suggest that relevant neurobiological mechanisms are indeed causally linked to focused attention and reduced MW.

The central-executive network (CEN; i.e., the frontoparietal network) includes dorsolateral prefrontal and parietal cortices (Fox et al., 2006), is involved in attentional processes. Therefore, it may also play an important role in MW and mindfulness. While spontaneous MW could be considered a form of failure in executive control processes (McVay and Kane, 2010; Seli et al., 2014), mindfulness practices are a form of an attentional regulatory training (Incagli et al., 2020), which can enhance executive functions and attentional control by promoting neuroplasticity and functional connectivity within CEN (Tang et al., 2015; Fox et al., 2014). Lastly, the salience network (SN), comprised of the anterior insula, the ventrolateral prefrontal cortex, and the anterior cingulate cortex, is considered to play a central role in detecting and prioritizing stimuli for attention (Seeley et al., 2007). The network is also involved in switching between the DMN and the CEN, facilitating the redirection of attention to relevant internal and external cues (Menon and Uddin, 2010). Experienced mindfulness meditators exhibit altered activation patterns in key regions of the SN involved in interoceptive awareness and self-referential processing (Farb et al., 2007; Fox et al., 2016). Therefore, the activities of CEN and SN seem to be closely linked to MW and mindfulness, in line with the triple network hypothesis (i.e., DMN, CEN and SN), which states that these networks are largely involved in cognitive and affective processes also when considering mental health and mental disorders (Menon, 2011; Langerbeck et al., 2023), mindfulness (Bremer et al., 2022; Kim et al., 2019), and MW (Kim and Lee, 2022). Interestingly, mindfulness increases connectivity from the left CEN to the SN, while MW increases connectivity from the DMN to the right CEN (Kim and Lee, 2022). Better understanding of the intricate connections among these networks, but also their relationship with mindfulness and MW (importantly, both in its spontaneous and deliberate aspects) are what is largely missing from current literature and warrants further investigation.

From a methodological perspective, previous connectivity studies involving these three networks usually focused on the strength of connections between different regions. However, recent evidence suggests that connectivity patterns are not static (such as those generally captured by the average indexes of frequency, amplitude, and phase), but may change over time (Calhoun et al., 2014; Chang and Glover, 2010). Therefore, we expanded the results of previous research (Preti et al., 2017) in the current study by taking into consideration the variability of the temporal dynamics of ICA based macro-networks (DMN, CEN, and SN). Temporal variability, i.e., the fluctuation of the blood-oxygenation-level-dependent (BOLD) neural signal over time (Zanella et al., 2022), has not been widely considered in the neuroimaging field so far. Indeed, the average rather than the fluctuations of the BOLD signal has usually been considered in previous studies, for example when task or resting-state conditions are considered. In addition, while temporal variability does not preserve the spatial distinction between nodes as done by other functional connectivity measures, its advantage is to consider the temporal properties of brain networks, without “collapsing across the temporal dimension” (Varley and Sporns, 2022). Indeed, considering these features allowed to discover that they are associated with different variables, such as age and cognitive states (Garrett et al., 2013), empathy and awareness (Stoica and Depue, 2020), emotional intelligence (Zanella et al., 2022) and emotion regulation (Guassi Moreira et al., 2019), functional network complexity and information integration (Garrett et al., 2010; Garrett et al., 2011; Vakorin et al., 2011).

This study aimed to extend the usage of the BOLD temporal variability in connection with MW and mindfulness questionnaires scores (measured as traits, not as states during resting-state) to add new evidence to the static brain features usually investigated, and thus shed new light on the neurocognitive mechanisms of these crucial personal tendencies.

Since the mind is expected to wander at rest (Poerio et al., 2017), we primarily focused on resting-state data to test whether BOLD temporal variability can predict self-reported trait mindfulness and MW by means of regression analysis. Both spontaneous and deliberate scores of MW were used separately in this analysis, unlike previous studies that did not distinguish between them, since some authors suggest stronger recruitment of DMN regions in spontaneous MW (Christoff et al., 2009; Christoff et al., 2016). As far as we know, no previous study has examined the predictability of MW and mindfulness from resting-state brain networks in one design. Since the brain activity at rest appears to facilitate spontaneous MW (Christoff et al., 2016), our hypothesis was that the temporal variability of DMN, among other networks, directly predicts spontaneous (but not deliberate) MW scores, which would be in turn inversely associated with mindfulness scores.

## 2 Materials and methods

### 2.1 Participants

From the Max Planck Institute sample (MPI-S) dataset (OpenNeuro database, accession number ds000221; Babayan et al., 2018), which includes behavioral, physiological and neuroimaging data from healthy people (Babayan et al., 2019), we selected participants according to the availability of the Five Facet Mindfulness Questionnaire (Baer et al., 2006), the spontaneous and deliberate mind wandering questionnaire (Carriere et al., 2013), their age range (20–45 years old, *m* = 27.1, *SD* = 5.08), right handedness, and negative drug test (when indicated). The final sample included 76 participants (female = 33).

### 2.2 Questionnaires

The spontaneous and deliberate MW scales (Carriere et al., 2013), each composed of 4 items, measure a person’s MW tendencies in everyday life distinguishing unintentional MW (e.g., I find my thoughts wandering spontaneously) and intentional MW (e.g., I allow my thoughts to wander on purpose) using a Likert-scale from 1 (almost never) to 5 (very often). The Five Facet Mindfulness Questionnaire (FFMQ; Baer et al., 2006) conceptualizes mindfulness as a construct with five distinct facets – observing (8 items), describing (8 items), acting with awareness (8 items), non-judging of inner experience (8 items), and non-reactivity to inner experience (7 items) using a 5-point Likert-scale (See Supplementary Table S1 for descriptives).

### 2.3 Data acquisition

Data was acquired on a 3 T Siemens Magnetom Verio Scanner during a 15-min resting-state (TR = 1,400 ms, TE = 30 ms, flip angle = 69°, echo spacing = 0.67 ms number of volumes = 657, voxel size = 2.3 mm) and a 8 min structural volume acquisition (TR = 5,000 ms, TE = 2.92 ms, TI1 = 700 ms, TI2 = 2,500 ms, FOV = 256 mm, voxel size = 1 mm isotropic).

### 2.4 Functional analysis

Resting-state data were preprocessed through the default pipeline for volume-based analysis of CONN software (Whitfield-Gabrieli and Nieto-Castanon, 2012)^1^ in MATLAB Version 9.4 (R2018a). This included: functional realignment and unwarping (coregistration to first scan using a least squares approach and a 6 parameter -rigid body- transformation), translation and centering, functional outlier detection (conservative settings-0.5 mm and 3 s.d. thresholds), functional direct segmentation and normalization (2 mm resolution), structural translation and centering, structural segmentation and normalization (2 mm resolution), and functional and structural smoothing (spatial convolution with Gaussian kernel 8 mm). Then we applied a component-based noise correction (CompCor), which involved linear regression and bandpass filtering (0.008–0.09 Hz) to remove unwanted motion and other artifact from the BOLD signal. After checking the data with quality assurance plots, independent component analysis (ICA) was applied to voxel-to-voxel analysis and group-ICA to extract the resting-state networks. ICA allows the extraction of patterns for naturally grouping functional connections of brain regions based on individual features of the participants rather than on *a priori* seeds or predefined masks (Kornelsen et al., 2020; Motoyama et al., 2019). This analysis based on Calhoun’s group-level ICA (Calhoun et al., 2001) included: variance normalization preconditioning (BOLD timeseries of each voxel are z-scored and PCA is applied, to ensure that components are not disproportionately influenced by signal amplitudes in certain regions or in some subjects), subject concatenation of BOLD signal data along a temporal dimension, group-level dimensionality reduction, fastICA for estimating independent spatial components, and group-ICA GICA1 back-projection for estimating individual subject-level spatial map (Nieto-Castanon, 2020; Whitfield-Gabrieli and Nieto-Castanon, 2012).

The ICA identified 20 networks (default settings; see Supplementary Figure S1). Through the CONN’s spatial-match-to-template function, networks best matching the triple network model (Menon, 2011) were selected for further analyses, according to the DICE similarity coefficient (DSC): DMN (*r* = 0.39), SN (*r* = 0.42), and CEN (*r* = 0.27). We also included sensorimotor (SMN, *r* = 0.50) and visual networks (VN, *r* = 0.15) for control analyses, as we expected significant associations of mindfulness and MW variables with the triple networks rather than with SMN and VN. However, MW could be related to visual imagery process (Cantone et al., 2021), and its scores could be associated with sensory networks’ temporal variability. To test this hypothesis, we included also these networks. Significant models were also tested by including other networks that potentially matched the selected ones with DSC > 0.25 (see Supplementary Table S2), to ensure that alternative results were excluded. Using the function ICA.Temporal.Components, the BOLD temporal variability (i.e., the fluctuation of the neural activity, measured as the standard deviation of the amplitude of the BOLD timeseries) of each network was extracted.

### 2.5 Linear regression

To determine for which temporal variability of the five above networks was predictive of MW and mindfulness scores, we examined the impact of each independent component’s (IC) BOLD signal variability on these psychological variables by conducting a linear multiple regression model with a backward elimination approach. The analyses were conducted separately for each of the seven dependent variables: spontaneous and deliberate MW and 5 FFMQ subscales with Bonferroni correction (*p*-value threshold = 0.05/7 = 0.007) and also included collinearity check (Variance Inflation Factor–VIF).

## 3 Results

### 3.1 Macro-networks contribution to mind wandering

**Figure 1:**
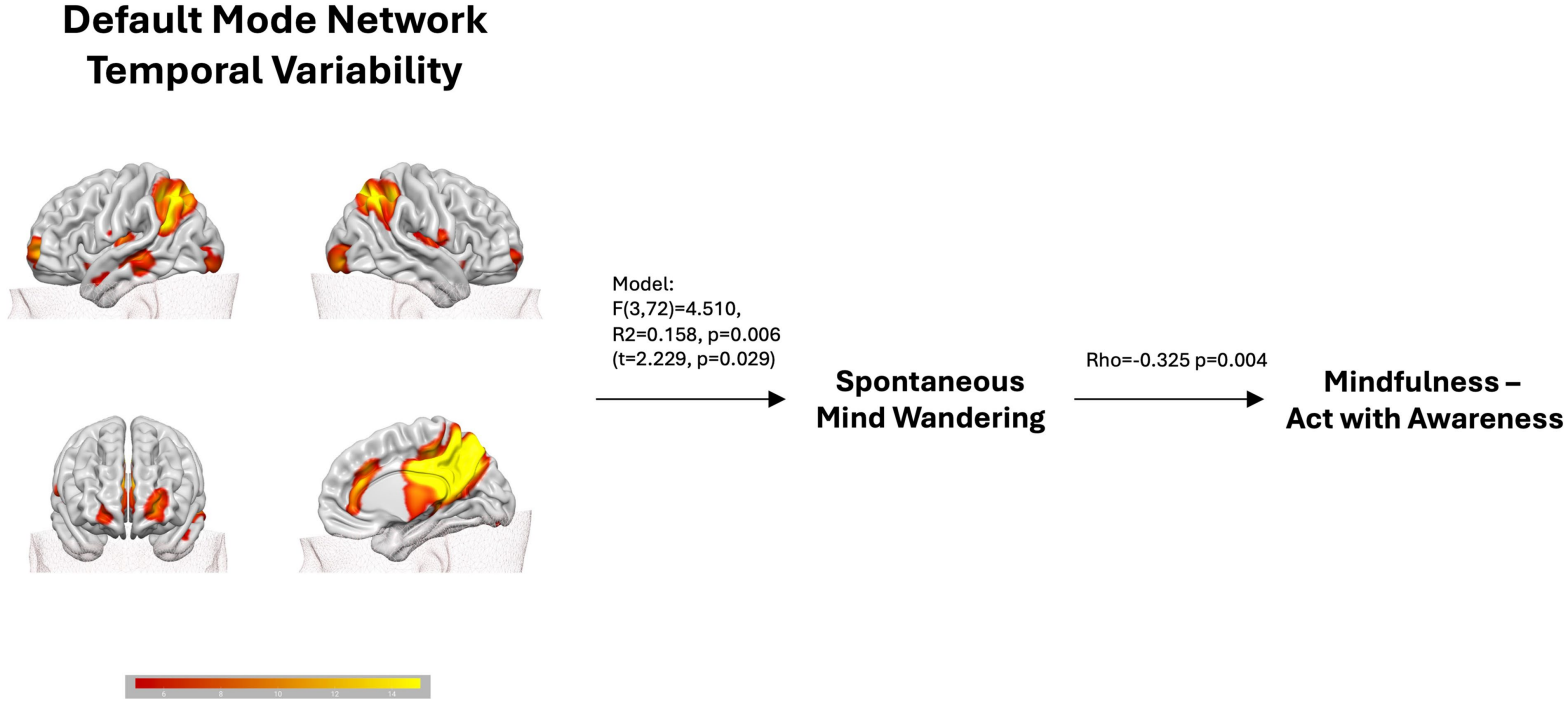
The figure shows that the Default Mode Network resting-state temporal variability predicts spontaneous mind wandering scores, which in turn are negatively correlated with the mindfulness FFMQ scale *Acting with awareness*.

The multiple regression with backward elimination method analysis of deliberate MW returned a significant winning model (*F* (1, 74) = 4.214, R^2^ = 0.055, *p* = 0.044) in which only the BOLD temporal variability of the DMN, IC16 (*t* = 2.053, *p* = 0.044), predicted deliberate MW. The corresponding regression equation was: dMW = 1.170 + 17.969*IC16. The areas of IC16 corresponded to the DMN (see Table 1; Figure 1 for IC16 data-driven identified brain areas).

**Table 1:** Brain areas included in the Default Mode Network, selected with a cluster significance level of *p* < 0.05 (FDR corrected) and of *p* < 0.001 at the voxel level.

Brain area	Clusters (x,y,z)	Size
Frontal Pole, Superior Frontal Gyrus	+ 40 + 38–14	69,047
Precuneous, Cingulate	+10–64 + 42	52,646
Supramarginal Gyrus	−64–32 + 42	1,626
Cerebellum	−64–54–46	671
Frontal Pole	+20 + 64–08	643
Cerebellum	+42–58–52	621
Cerebellum	−08–46–48	604
Middle Frontal Gyrus	−32 + 06 + 56	498
Middle Temporal Gyrus	+60–40–16	171
Middle Temporal Gyrus	−64–14–26	158
Middle Frontal Gyrus	+32 + 24 + 48	71
Middle Frontal Gyrus	−24 + 36 + 30	68
Cerebellum	+26–44–58	49

The backward multiple regression analysis of spontaneous MW returned a significant winning model (*F* (3, 72) = 4.510, R^2^ = 0.158, *p* = 0.006) in which the BOLD temporal variability across the DMN, IC16 (*t* = 2.229, *p* = 0.029), significantly predicted spontaneous MW. The model included also the SN, IC2 (*t* = −1.896, *p* = 0.062), and the VN, IC15 (*t* = 1.893, *p* = 0.062). All predictors show VIF < 1.04, meaning absence of collinearity. Although they contributed to the winning model, explaining part of the variability, they did not reach significance. The corresponding regression equation was: sMW = 0.644–10.371*IC2 + 18.958*IC16 + 13.595*IC15.

Following the correction for multiple comparisons, only the model of spontaneous MW remained statistically significant. No change occurs even if other networks potentially matching those analyzed (according to DSC) are included (see Supplementary Table S2).

Additionally, we conducted a seed-to-voxel analysis which confirmed and expanded our main results by showing a significant spontaneous MW modulation of the connectivity between the DMN and the right frontal pole (FDR-corrected *p* = 0.012; see Supplementary materials).

### 3.2 Macro-networks contribution to mindfulness

The backward multiple regression analyses did not return any significant model associated with the FFMQ subscales: *describing* (F (1, 74) = 1.874, R^2^ = 0.025, *p* = 0.175), *observing* (F (1, 74) = 0.256, R^2^ = 0.003, *p* = 0.614), *acting with awareness* (F (1, 74) = 2.510, R^2^ = 0.033, *p* = 0.117), *non-judging* (F (1, 74) = 3.820, R^2^ = 0.049, *p* = 0.054), *non-reactivity* (F (1, 74) = 3.187, R^2^ = 0.041, *p* = 0.078).

### 3.3 Relationships between MW and mindfulness

Correlations were used to check whether MW subscales were associated with FFMQ subscales (see Table 2). We found a significant negative relationship between spontaneous MW and the acting with awareness FFMQ subscale (rho = −0.325, *p* = 0.004) and a significant positive relationship between deliberate MW and the observing FFMQ subscale (rho = 0.260, *p* = 0.024). However, only the first correlation survived the Bonferroni correction (*p*-value threshold = 0.05/10 = 0.005). This holds true even when correcting for the DMN temporal variability (rho = −0.328, *p* = 0.004). Spontaneous and deliberate mind wandering showed a significant correlation (*r* = 0.463, *p* < 0.001).

**Table 2:** Spearman’s correlations between MW and mindfulness FFMQ subscales.

Variable		MW_ delib	MW_ spont
FFMQ observing	Rho	0.260*	−0.125
	*p*-value	0.024	0.283
FFMQ describing	Rho	0.066	−0.096
	*p*-value	0.572	0.412
FFMQ acting with awareness	Rho	−0.060	−0.325
	*p*-value	0.605	0.004**
FFMQ non-judging	Rho	−0.167	−0.105
	*p*-value	0.149	0.367
FFMQ non-reactivity	Rho	0.041	−0.181
	*p*-value	0.723	0.118

*p < 0.05; **p < 0.01; ***p < 0.001.

### 3.4 Effects of age and gender

We performed *t*-tests and correlations to evaluate the effects of gender and age on FFMQ, and MW scores, and temporal variability of the DMN (IC16). See Table 3. For the FFMQ *non-judging* subscale, it showed that males had higher scores than females (*t* = −2.175, *p* = 0.033). Age was negatively correlated with the DMN variability (rho = −0.30, *p* = 0.008). This holds true even when correcting for spontaneous MW (rho = −0.33, *p* = 0.003).

**Table 3:** Gender and age effects. Independent samples Student’s *t*-test of gender differences and Spearman’s correlations with age are shown.

Variable	Gender		Age	
IC16 (DMN) temporal variability	t	1.498	Rho	−0.301**
	*p*-value	0.138	*p*-value	0.008
FFMQ observing	t	1.745	Rho	0.043
	*p*-value	0.085	*p*-value	0.710
FFMQ describing	t	1.699	Rho	−0.046
	*p*-value	0.094	*p*-value	0.690
FFMQ acting with awareness	t	−1.563	Rho	−0.223
	*p*-value	0.122	*p*-value	0.052
FFMQ non-judging	t	−2.175	Rho	0.049
	*p*-value	0.033	*p*-value	0.676
FFMQ non-reactivity	t	−0.988	Rho	−0.063
	*p*-value	0.327	*p*-value	0.587
MW deliberate	t	−0.256	Rho	0.046
	*p*-value	0.799	*p*-value	0.691
MW spontaneous	t	1.262	Rho	0.094
	*p*-value	0.211	*p*-value	0.419

*p < 0.05; **p < 0.01; ***p < 0.001.

## 4 Discussion

This study aimed to employ BOLD temporal variability measurements to evaluate the relationships of MW and mindfulness scores obtained from self-reports, and specific resting-state networks collected during a 15-min resting condition. Additionally, we wanted to distinguish the specific contribution of spontaneous and deliberate MW in these relationships. Consistent with our hypothesis, results show that the DMN’s temporal variability directly predicts spontaneous, but not deliberate MW, which is in turn negatively associated with mindfulness (*acting with awareness* FFMQ subscale; see Figure 1). The temporal variability indexes of the other examined networks (CEN, SN, VN, SMN) did not emerge as significant predictors. We also observed that mindfulness scores, as opposed to MW scores, were not directly predicted by temporal variability of the DMN or other brain networks. Although the positive effects of mindfulness can be measured in brain structures and functions through different methods (see Tang et al., 2015 for a review), in this study we relied on resting-state data that was acquired when participants were not engaged in any specific activity. Therefore, it is reasonable to assume that in such condition the DMN was predominantly active, and MW was taking place (Buckner and Carroll, 2007; Moran et al., 2006; Raichle et al., 2001). The connection between DMN and other brain networks with mindfulness scores could possibly be more difficult to establish in resting-state conditions using the temporal variability index of brain network activity as it was employed in the current study.

The temporal variability is a relatively new index previously associated with variables such as individuals’ age and cognitive states (Garrett et al., 2013) and emotional features (Guassi Moreira et al., 2019; Sorella et al., 2022; Zanella et al., 2022). Our data extend and support this evidence showing a negative correlation between age and the DMN variability (Rho = −0.30, *p* = 0.008). This is in line with the effects of aging on the DMN (Mevel et al., 2013) and with previous observations on the naturally occurring change in macro-networks during development (Jones et al., 2011). Besides the effects on age, our main result extends previous findings by showing a positive association between the DMN temporal variability and spontaneous MW rather than deliberate MW or mindfulness. This means that the higher the variability of the DMN during rest, the higher the tendency to experience self-reported spontaneous MW. This suggests that higher number of MW episodes implies greater variability of cognitive states in which the mind spontaneously wanders on and off, resulting in higher variability of the DMN. According to our results, MW could be the experiential counterpart of the DMN (Mason et al., 2007; McKiernan et al., 2006) since they share many similarities, with both “opposing” to external perception and showing anti-correlation with brain regions involved in external sensory processes (Smallwood and Schooler, 2015). In addition, a meta-analysis of functional neuroimaging studies identified key DMN areas consistently involved in MW (Fox et al., 2015), while a study by Mason et al. (2007) found increased DMN recruitment during periods of frequent MW. The results are not surprising given that MW includes different mental processes such as autobiographical memory retrieval, future planning, and self-referential processing, which primarily recruit DMN regions (Smallwood and Schooler, 2015).

In the context of our study, it was important to clarify the phenomenology of MW by distinguishing deliberate (or intentional) and spontaneous (or unintentional) processes. Interestingly, it has been proposed that the DMN seems to be recruited during spontaneous MW, whereas meta-awareness and deliberate constraints of thinking are relatively weak (Christoff et al., 2016). This notion is confirmed by our results, according to which spontaneous, but not deliberate, MW can be predicted by the temporal variability of DMN. The phenomenology of MW should also be considered in relation to mindfulness. In line with previous evidence (Carriere et al., 2013), we found that only spontaneous (but not deliberate) MW is negatively associated with the *acting with awareness* scale of the FFMQ. Indeed, spontaneous MW typically involves a decoupling of attention from the external environment (Schooler et al., 2011), leading to the activation of self-referential thoughts, memories, and future-oriented concerns (Smallwood and Schooler, 2015) associated with the DMN (Christoff et al., 2009, 2016). Instead, acting with awareness, which is shown to be cultivated in experienced meditators whose DMN areas may be relatively deactivated (Brewer et al., 2011), entails sustained attention to present-moment experiences (Brown and Ryan, 2003). These two states (spontaneous MW and acting with awareness) could therefore represent quite opposite cognitive modes wherein MW could undermine the ability to act with awareness by disrupting attentional focus, impairing cognitive control, and promoting behavioral automaticity (Mrazek et al., 2013). On the other hand, deliberate MW was positively correlated with the FFMQ *observing* subscale, thus highlighting the complexity of the MW construct and the fact that the intentional aspects of this construct may not act in an opposing fashion to mindfulness states. For this reason, as suggested by some authors (Cantone et al., 2021; Seli et al., 2014), it is critically important to continue to distinguish the different phenomenological facets of MW, as to increase knowledge of this phenomenon as rigorously as possible. A recent study we conducted confirms the importance to explore the dynamic and multifaceted characteristics of mindfulness and MW, showing congruent findings on the role of acting with awareness as a mediator between spontaneous MW and structural brain networks (Chang et al., 2024).

Finally, in our study no significant result emerged when considering the other examined networks (CEN, SN, VN, and SMN). Indeed, for SMN and VN, there seems to be no consistent association between MW and sensory activity (Christoff et al., 2009) except for pain perception (Kucyi et al., 2013). Quite on the contrary, it has been proposed that MW is characterized by a decoupling from perceptual stimuli, which may attenuate activity in the sensory cortices (Schooler et al., 2011). Although a recent study found that the connectivity between the insula and the sensory cortex mediates the relationship between mindfulness and MW scores (He et al., 2024), our study extends prevailing evidence for the lack of connections between the sensory networks and MW, while relying for the first time on the temporal variability of resting-state networks. Moreover, previous neuroimaging studies suggest that the CEN would be negatively associated with MW episodes if they are considered as failure in executive control (Seli et al., 2014). In a similar fashion, the SN (e.g., the insula as a hub of interoceptive awareness) could have been expected to be involved with mindfulness in the present study (Farb et al., 2007). In contrast with this evidence obtained from studies based on brain connectivity and functional activation analysis, the present findings showed no significant relationship between networks such as the SN and CEN with MW or mindfulness facets in terms of the temporal variability index of these networks at rest.

In summary, we found that the temporal variability can be effectively used to establish the DMN predictability of spontaneous MW scores, which is in turn negatively associated with the mindfulness scale *acting with awareness*. No significant relationship was found in relation to the temporal variability of other brain networks at rest. The present study presents novel findings, but it also has certain limitations. One limitation is that the DMN identified via ICA exhibits some deviations from commonly referenced group network maps (Yeo et al., 2011; Power et al., 2011; Gordon et al., 2017). Specifically, our network includes a medial PFC cluster positioned more dorsally and posteriorly, a lateral parietal cluster extending dorsally, and clusters extending into insular and visual regions. These deviations may hint at overlaps with other networks, such as the CEN, SN or VN. However, the spatial-match-to-template function demonstrates significant correspondence solely with the DMN (see Supplementary Table S2), underscoring that the primary network identified aligns with the DMN. Although in other studies (Schöpf et al., 2010; Shirer et al., 2011) ICA networks have better approximated canonical networks, discrepancies are not unique to this study and have also been reported in similar analyses using ICA (Motoyama et al., 2019; Sorella et al., 2022). The ICA identifies naturally occurring patterns of functional connectivity in the participants studied, without being constrained by predefined boundaries. This approach captures individual variability in brain networks, which can offer richer insights but may also result in differences in spatial localization compared to more standardized templates. While these differences might appear as limitations at first, they also highlight one of ICA’s key strengths.

Additionally, our analysis focused on a specific mental condition of rest (i.e., resting-state). Future studies may consider different cognitive states through task-specific experimental designs (e.g., attentional tasks or meditation sessions) to possibly find new evidence for the temporal variability of other brain networks (e.g., SN and CEN). Longitudinal studies involving mindfulness training could also provide a better measure of changes in the temporal variability of various resting-state networks in relation to personal changes in MW tendencies and mindfulness abilities.

## Data Availability

Publicly available datasets were analyzed in this study. This data can be found at: https://openneuro.org/datasets/ds000221/versions/00002, OpenNeuro accession number ds000221.

## Footnotes

^1^ https://web.conn-toolbox.org
