# Supplementary material for: Resting-state BOLD temporal variability of the default mode network predicts spontaneous mind wandering, which is negatively associated with mindfulness skills
Source: Front Hum Neurosci. 2025 Jan 22;19:1515902. doi: 10.3389/fnhum.2025.1515902 (PMC11794827; doi:10.3389/fnhum.2025.1515902)
Supplement: Supplementary file 1 [file Data_Sheet_1.docx]

| **Table S1. Descriptive Statistics** of the questionnaires and the independent components used in the analyses. | | | | | | | | | | | | | | | | | | | | | | | | | |
| --- | --- | --- | --- | --- | --- | --- | --- | --- | --- | --- | --- | --- | --- | --- | --- | --- | --- | --- | --- | --- | --- | --- | --- | --- | --- |
|  | | FFMQ_observe | | FFMQ_describe | | FFMQ_act_awareness | | FFMQ_nonjudge | | FFMQ_nonreact | | MW_delib | | MW_spont | | SN | | CEN | | VN | | DMN | | SMN | |
| Mean |  | 22.171 |  | 28.842 |  | 16.724 |  | 20.789 |  | 18.882 |  | 3.487 |  | 3.105 |  | 0.171 |  | 0.152 |  | 0.131 |  | 0.129 |  | 0.132 |  |
| Std. Deviation |  | 3.407 |  | 5.822 |  | 2.822 |  | 4.585 |  | 3.536 |  | 0.958 |  | 0.956 |  | 0.019 |  | 0.017 |  | 0.015 |  | 0.012 |  | 0.016 |  |
| Minimum |  | 12.000 |  | 14.000 |  | 12.000 |  | 12.000 |  | 6.000 |  | 1.000 |  | 1.000 |  | 0.131 |  | 0.119 |  | 0.101 |  | 0.101 |  | 0.099 |  |
| Maximum |  | 29.000 |  | 40.000 |  | 22.000 |  | 29.000 |  | 29.000 |  | 5.000 |  | 5.000 |  | 0.219 |  | 0.199 |  | 0.167 |  | 0.155 |  | 0.175 |  |
|  | | | | | | | | | | | | | | | | | | | | | | | | | |

FFMQ – Five Facet Mindfulness Questionnaire; observe – observing subscale; describe – describing subscale; act_awareness – acting with awareness subscale; nonjudge – non judging of inner experience subscale; nonreact – non reactivity to inner experience subscale. MW – spontaneous and deliberate mind wandering scales; delib – deliberate subscale; spont – spontaneous subscale. SN – Salience Network temporal variability; CEN – Central Executive Network temporal variability; VN – Visual Network temporal variability; DMN – Default Mode Network temporal variability; SMN – Sensorimotor Network temporal variability.

**Table S2. Spatial Overlap of Suprathreshold areas between ICA-based identified networks and standard maps (DICE similarity coefficient).** The spatial-march-to-template function of CONN shows the best three matches for each network template among the different ICs, and the best three match for each IC among the different network templates. The table shows all available information: “/” means DICE coefficient <0.001 or not indicated (only the best three matches to each IC are reported in CONN). Included networks are: DMN – default mode network; SN – salience network; CEN – central executive network; SMN – sensorimotor network; VN – visual network.

|  | | DMN | | SN | | CEN | | SMN | | VN | |  |  |
| --- | --- | --- | --- | --- | --- | --- | --- | --- | --- | --- | --- | --- | --- |
| IC1 |  | / |  | / |  | / |  | / |  | / |  |  |  |
| IC2 |  | / |  | 0.416 |  | / |  | / |  | / |  |  |  |
| IC3 |  | / |  | / |  | / |  | / |  | / |  |  |  |
| IC4 |  | 0.145 |  | / |  | / |  | / |  | 0.009 |  |  |  |
| IC5 |  | / |  | / |  |  | 0.266 | / |  | / |  |  |  |
| IC6 |  | / |  | 0.072 |  | / |  | 0.148 |  | / |  |  |  |
| IC7 |  | / |  | / |  |  | 0.17 | / |  | / |  |  |  |
| IC8 |  | / |  | 0.008 |  | / |  | 0.09 |  | / |  |  |  |
| IC9 |  | 0.094 |  | / |  | / |  | / |  | 0.542 |  |  |  |
| IC10 |  | / |  | / |  | / |  | / |  | / |  |  |  |
| IC11 |  | 0.359 |  | / |  | / |  | 0.049 |  | / |  |  |  |
| IC12 |  | 0.039 |  | / |  |  | / | / |  | 0.442 |  |  |  |
| IC13 |  | 0.052 |  | 0.062 |  |  | / | / |  | / |  |  |  |
| IC14 |  | / |  | / |  |  | / | 0.484 |  | / |  |  |  |
| IC15 |  | / |  | / |  |  | / | / |  | 0.582 |  |  |  |
| IC16 |  | 0.387 |  | / |  |  | 0.066 | / |  | / |  |  |  |
| IC17 |  | / |  | / |  |  | / | / |  | / |  |  |  |
| IC18 |  | / |  | / |  |  | / | 0.264 |  | 0.020 |  |  |  |
| IC19 |  | 0.082 |  | / |  |  | / | / |  | 0.145 |  |  |  |
| IC20 |  | / |  | / |  |  | / | 0.505 |  | / |  |  |  |

**Figure S1.** The figure shows the 20 Independent Components (i.e., independent brain networks) identified by our analysis. DMN is IC16; SN is IC2; FPN is IC5; VN is IC15; SMN is IC20.

**Seed-to-voxel analysis**

To confirm our results on the role of the DMN temporal variability in the prediction of spontaneous MW scores, we decided to rely on a widely used measure, i.e., seed-based connectivity metrics. This measure characterizes the connectivity patterns with a pre-defined seed or ROI (Region of Interest). Indeed, we were interested in the connectivity patterns between the DMN nodes and the rest of the brain when considering the modulation of spontaneous MW scores.

The preprocessing steps were the same of the ICA, described in the main text of the paper. Then, instead of applying ICA we conducted a seed-to-voxel analysis of the nodes of the DMN (medial prefrontal cortex, posterior cingulate cortex, left and right parietal cortices) with the rest of the brain, considering the modulatory effect of spontaneous MW scores. Seed-based connectivity maps (SBC) were estimated characterizing the patterns of functional connectivity with 164 HPC-ICA networks^[1]^ and Harvard-Oxford atlas ROIs^[2]^. Functional connectivity strength was represented by Fisher-transformed bivariate correlation coefficients from a weighted general linear model (weighted-GLM^[3]^), defined separately for each pair of seed and target areas, modeling the association between their BOLD signal timeseries. Then, group-level analyses were performed using a General Linear Model (GLM). For each individual voxel a separate GLM was estimated, with first-level connectivity measures at this voxel as dependent variables, and subject-level identifiers as independent variables (i.e., mindfulness and mind wandering questionnaires scores; see the main text for further details). Voxel-level hypotheses were evaluated using multivariate parametric statistics with random-effects across subjects and sample covariance estimation across multiple measurements. Inferences were performed at the level of individual clusters (groups of contiguous voxels). Cluster-level inferences were based on parametric statistics from Gaussian Random Field theory. Results were thresholded using a combination of a cluster-forming p < 0.001 voxel-level threshold, and a familywise corrected p-FDR < 0.05 cluster-size threshold.

Results show that spontaneous MW scores significantly modulate the connectivity between the DMN and the right frontal pole (MNI coordinates: +28 +52 +06). This result is in line with the main result of the study and further confirms the involvement of the DMN in spontaneous MW.


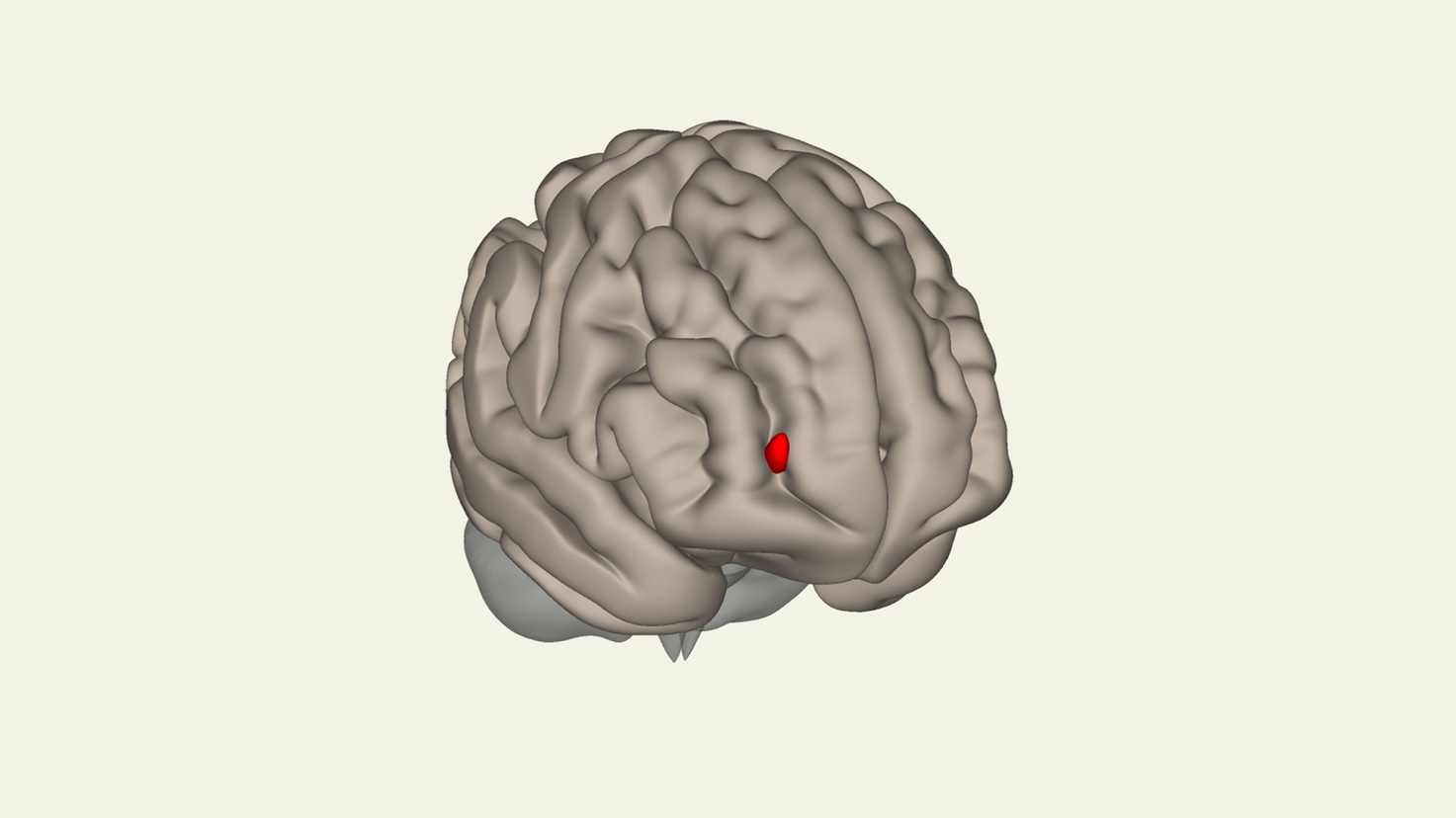


**Figure S2.** The figure shows the results of the seed-to-voxel analysis. Results show that DMN nodes are significantly connected with the right frontal pole (in red) when considering the effect of spontaneous MW scores.

References:

^[1]^ Nieto-Castanon, A. & Whitfield-Gabrieli, S. (2018). CONN functional connectivity toolbox: RRID SCR_009550, release 18. doi:10.56441/hilbertpress.1818.9585.

^[2]^ Desikan R.S., SÃ©gonne F., Fischl B., Quinn B.T., Dickerson B.C., Blacker D., Buckner R.L., Dale A.M., Maguire R.P., Hyman B.T., Albert M.S., & Killiany R.J. (2006) An automated labeling system for subdividing the human cerebral cortex on MRI scans into gyral based regions of interest. Neuroimage 31(3):968-980

^[3]^ Nieto-Castanon, A. (2020). Functional Connectivity measures. In Handbook of functional connectivity Magnetic Resonance Imaging methods in CONN (pp. 26â€“62). Hilbert Press.
